# Oral care considerations for people with cystic fibrosis: a cross-sectional qualitative study

**DOI:** 10.1038/s41405-023-00136-w

**Published:** 2023-03-11

**Authors:** Niamh Coffey, Fiona O’ Leary, Francis Burke, Barry Plant, Anthony Roberts, Martina Hayes

**Affiliations:** 1 Department of Restorative Dentistry, Cork University Dental School and Hospital, University College Cork, Cork, T12 E8YV Ireland; 2 Adult Cystic Fibrosis Centre, Cork University Hospital, Wilton, Cork, T12 DFK4 Ireland; 3 Department of Restorative Dentistry, Dublin Dental University Hospital, Lincoln Place, Dublin, Ireland

**Keywords:** Dental anxiety and phobia, Special care dentistry

## Abstract

**Objectives:**

To investigate the attitudes of adults with Cystic Fibrosis (CF) towards dental attendance and any perceived barriers to treatment.

**Methods:**

A cross sectional survey in the form of a structured, anonymous questionnaire was used to obtain information regarding adults with CF’s feelings towards dentists and dental treatment. The final version of the questionnaire was based on a collaborative effort between researchers at Cork University Dental School and Hospital and Cystic Fibrosis (CF) patient advocates from CF Ireland. Participants were recruited via CF Ireland’s mailing list and social media channels. The responses underwent descriptive statistical analysis and inductive thematic analysis.

**Results:**

A total of 71 people (33 Male: 38 Female) over the age of 18 living with CF in the Republic of Ireland responded to the survey. 54.9% of respondents were unhappy with their teeth. 63.4% felt that CF had an impact on oral health. 33.8% were anxious about attending their dentist. Respondents believed that CF has impacted on their oral health due to the medications and dietary requirements involved, as well as tiredness and other side effects of CF. Reasons for being anxious about attending the dentist included cross infection concerns, issues with the dentist, with tolerating treatment, and with the teeth themselves. Respondents wanted dentists to be aware of the practicalities of dental treatment for people with CF, especially their discomfort with lying back. They also want the dentist to be aware of the impact that their medication, treatment and diet has on their oral health.

**Conclusions:**

Over one third of adults with CF reported anxiety about attending the dentist. Reasons for this included fear, embarrassment, cross infection concerns and problems with treatment, especially being in the supine position. Adults with CF want dentists to be aware of the impact that CF can have upon dental treatment and oral health care.

## Introduction

Cystic Fibrosis (CF) is an autosomal recessive genetic condition, resulting from mutations in the cystic fibrosis transmembrane conductance regulator (CFTR) gene on chromosome 7 [1] Its dysfunction results in abnormal transport of Chloride and Bicarbonate ions, leading to thick viscous secretions in multiple organ systems. This can result in diabetes, osteoporosis, liver disease, chronic kidney disease, and gastro-intestinal issues, as well as serious respiratory problems, with progressive bronchiectasis and recurrent infective pulmonary exacerbations being the hallmark of CF lung disease [2]. Lung transplantation is recommended for individuals with advanced lung disease [3]. Due to earlier diagnosis and improved therapies, the life expectancy of people with CF (PWCF) has greatly improved, from a median survival age of 11 years in 1978 to between 44 and 53 years in 2019 [4]. Due to the fact that CF has changed from an almost exclusively paediatric disease to a disease of adulthood, the transition to adult care is now an important aspect of CF patient management [5]. The medical care of PWCF is complex, with treatment aiming to control the symptoms, reduce complications and reduce the severity of different manifestations of CF. Oral care considerations include the long-term use of antibiotics (which may be inhaled or nebulised), bisphosphonates for osteoporosis, presence of CF related diabetes, gastro-oesophageal reflux disorder, and malnutrition which may be treated with Oral Nutritional Supplements with a high sugar content [6–10].

The oral health status of individuals with CF is not yet fully understood. A 2020 systematic review showed that the majority of studies show better oral hygiene, with lower levels of gingivitis and plaque among PWCF than controls [11], with fewer studies showing increased gingivitis and higher levels of plaque and calculus. It is thought that the long-term use of broad-spectrum antibiotics may have a preventative effect against the development of periodontal disease [12].

A number of studies have investigated the link between CF and Developmental Defects of Enamel (DDEs) and the majority of these report an increased level of DDEs in the CF population [13–18]. It is hypothesised that this may be due to metabolic or nutritional disturbances, long term antibiotic use and pancreatic enzyme use [14, 18]. Interestingly, Abu-Zahra et al. also found that the severity of enamel defects increased with the number of surgeries the patient had undergone [15].

There has been significant research into the relationship between CF and dental caries experience, however, no clear consensus can be formed. Despite the fact that people with CF have been hypothesised to be more at risk of caries due to a high-calorie diet, sugar containing oral nutritional supplements, gastro-oesophageal reflux disorder (GORD), increased levels of *streptococcus mutans* and DDEs [14, 19], the majority of studies found that there was a lower caries experience in people with CF [12, 15–18, 20–23]. However, some studies found that caries experience in people with CF increased with age [24, 25], and, furthermore, studies that included adults, or were limited to adult participants found a higher caries experience in study groups [13, 26, 27].

Studies have shown that people with special medical care needs may be less likely to access dental care [28] and a recent report has shown that people with Cystic Fibrosis may attend dental practices less frequently than is recommended [29].

There has been no prior research into the attitudes of adults with CF regarding dental attendance. This study was undertaken to investigate concerns adults with CF may have regarding dental attendance and dental treatment, and to identify methods to improve service provision for these individuals.

## Aims

This study seeks to investigate the attitudes of adults with CF towards dental attendance and any perceived barriers to treatment.

## Methodology

### Study design

A cross sectional survey was carried out in accordance with the World Medical Association Declaration of Helsinki and received Ethical Approval from the Clinical Research Ethics Committee of the Cork Teaching Hospitals (ECM 03/2022 PUB).

The inclusion criteria were people over the age of 18 years with a diagnosis of CF. The exclusion criteria were people without Cystic Fibrosis, or people with Cystic Fibrosis under the age of 18. All respondents gave written consent to take part, declaring that they understood they were under no obligation to complete the questionnaire and that they consented to data collection. Participants were recruited via Cystic Fibrosis Ireland’s mailing list and social media channels.

### Questionnaire

A structured, anonymous online questionnaire, with open-ended questions, was used to obtain information regarding their feelings towards dentists and dental appointments. Patient involvement was sought from patient advocates from CF Ireland, with whom the questionnaire was trialed, and amendments made based on their feedback. The final version of the questionnaire was based on a collaborative effort between researchers at Cork University Dental School and Hospital and CF patient advocates from CF Ireland. Data was collected from May to August 2020. A copy of the questionnaire is included in Appendix I.

### Data analysis

Descriptive statistical analysis of the quantitative questions was completed using IBM SPSS (v26; SPSS Inc., Chicago, IL, USA). The qualitative portion underwent thematic analysis as described by Braun and Clarke [30] (2006). The analysis was inductive in that there is no previous study in this area and, therefore, the data collected determined the themes generated. Selected quotations and related themes can be seen in Appendices II-IV.

## Results

A total of 71 adults with CF responded to the survey.

The quantitative responses and descriptive statistical analysis are summarised in Table 1.

**Table 1:** Respondent Profile.

Variable	Category	*N*	%
Gender	Male	33	46.5
	Female	38	53.5
Level of education completed	Primary Level	3	4.2
	Secondary Level	17	23.9
	Third Level	48	67.6
Employment	Full-/part- time employment/self-employed	43	60.6
	Unemployed	25	35.2
	Prefer not to say	3	4.2
Natural teeth	20 or more	64	90.1
	10-19	4	5.6
	Don’t know	3	4.2
Denture	Wears denture	0	0
	No Denture	71	100
Are you happy regarding the appearance of your teeth?	Yes	30	41.3
	No	39	54.9
	Declined to answer	2	2.8
Dental attendance	Regular attender	42	59.1
	Irregular attender	29	40.8
Reason for last dental visit	Routine check-up/consultation	39	54.9
	Pain/trouble with teeth gums or mouth	21	29.6
	Treatment	8	11.3
Do you think that CF has impacted your oral health?	Yes	45	63.4
	No	13	18.3
	Don’t know/Declined to answer	13	18.3
Anxious/worried about attending dentist?	Yes	24	33.8
	No	45	63.4
	Don’t Know	2	2.8
Do you make your dental practice aware of your CF status?	Yes	25	35.2
	No	19	26.8
	Dental practice already aware	27	38

The majority of respondents had a high level of education (67.6% having 3rd level education) and were employed or self-employed (60.6%). They retained the majority of their teeth and no respondent had a denture. Despite this, the majority of patients (54.9%) were unhappy with the appearance of their teeth. 40.8% of people were irregular attenders and nearly 30% said the reason for their last dental visit was “pain or trouble with teeth, gums or mouth”. The majority (63.4%) of patients felt that CF had an impact on their oral health. 33.8% were anxious about attending the dentist. 25.2% of respondents made the dental practice aware of their CF status before attending, with a further 38% reporting that the dental practice was already aware.

When asked if they think dentists should be part of the multidisciplinary team, 54.9% said “Yes”, 32.4% said “Maybe” and only 12.7% said “No”.

### Qualitative data

The following questions underwent thematic analysis:

1. *Do you believe that CF has impacted on your oral health in any way?*
2. *Are you anxious/worried about going to the dentist? If yes, why?*
3. *What do you think is important for the dentist to know about CF?*

#### *Question 1:*

*Do you believe that CF has impacted on your oral health in any way?*

The majority of respondents (63.4%) believed that CF did have an impact on their oral health. The major themes that emerged from this question, in decreasing frequency was:

1. Impact of medications (especially antibiotics)
2. Tiredness
3. Diet
4. Side effects of CF

The main CF-related factor that respondents mentioned was the impact of medications- concerns ranged from nausea/vomiting to discolouration, to dry mouth. Many blamed antibiotics in particular as the cause for “*staining*”, “*lost enamel*” and “*weakened teeth*”.

Tiredness/lack of energy also had an impact on their oral health. Many mentioned being “*too tired to brush*”, “*no energy to brush*”, with one respondent noting “*When I’ve been very sick it’s hard to get the energy to brush your teeth, I remember having to ask my mum to brush them for me”*.

Another area where CF impacted oral health was diet. Many respondents noted an increase in amount of sugar needed: “*to get calories in*”, “*just over eating sweet things when sick and no real appetite for large meals*”, and being “*addicted to sugar*”. One respondent noted that they had an increased need for sugary food since developing CF related diabetes (CFRD) *“Since developing CFRD, I eat a lot more jellies and drink orange juice now to treat lows [hypoglycaemic episodes]*.

Other systemic effects of CF also had an impact on their dental health, such as “*tummy issues causing bad breath and erosion*” and missing dental appointments due to being unwell.

#### *Question 2:*

*Why are you anxious/worried about going to the dentist?*

Five major themes emerged from this question, in descending order of frequency, these were:

1. Issues with the dentist
2. Cross-infection concerns
3. Problems with treatment
4. Problems with teeth
5. CF related problems.

Dentist-related problems included fear of dentists themselves, fear of being judged by the dentist, with some respondents noting “*I have previously been treated with lack of understanding from dentists/hygienists*” and ““*I am always in trouble when I attend”*.

Another aspect of dental visits that concerned adults with CF was cross-infection control, with many respondents mentioned increased anxiety since the Covid-19 pandemic *“I did not feel this way before but I do now in light of Covid-19”*. Some respondents also mentioned concern at being so “*close to the sink at dentist chair*”. This is due to the perception that there may be stagnant water or saliva in the sink [spittoon] which could pose a cross-infection risk to the individual.

A practical issue that caused anxiety in adults with CF was dental treatment, with many expressing difficulty in lying in the supine position that is usually employed during a dental examination- “*I find it difficult to be in a flat position as it affects my breathing. It’s also very difficult not to cough”*. Some respondents reported dental problems causing anxiety surrounding dental appointments: “*My teeth are all wearing away at the bottom the past few years and I feel my teeth are going to break soon”, “I just feel embarrassed about my teeth”*.

CF itself had an impact on people’s anxiety regarding dental attendance, with one individual noting that they *“Feel sick enough without there being another thing wrong with my teeth”*.

#### *Question 3:*

*What do you think is important for the dentist to know about CF?*

Four major themes emerged from this question. In descending order of frequency, they were:

1. Practicalities of dental treatment
2. Impact of medication/treatment/diet
3. Impact of CF itself
4. Cleanliness of surgery/cross-infection control.

The main concerns adults with CF had were the practicalities of dental treatment, namely keeping the patient upright and allowing them to have breaks during treatment. The vast majority of respondents said some variant of this, e.g., *“it is often difficult for people with CF to lie down and not cough for long periods of time*”, “*would have liked option to sit more upright during prolonged treatment, felt this was where I was exposed to risk of aspiration”, “I can’t lie flat or sometimes breathe through my nose quick enough*”. On a positive note, some respondents noted that their dentist/hygienist already accommodates this: “*My dentist is very good and tilts chair upright and also pauses when I indicate I need to cough. It slows process but he is very understanding and allows time*”, “*Extra breaks may be needed, my dentist/hygienist are good when it come to that!*”.

Another issue that adults with CF wanted dentists to be aware of is the impact that medication, treatment and diet can have on their oral health. A concern is that dentists are not aware or not understanding of the effects that CF-related medication and diet has on their oral health. They want dentists to be aware of “*the full extent of the treatment required that may impact the teeth, gums and tongue”, “the effect different meds have on oral hygiene”, “before lecture on oral hygiene, sweets etc.- CF patients need [them] to maintain weight”*. Another overlapping theme that emerged was for the dentists to be aware of CF itself and the impact that has on the individual: “*the impact it [CF] has on our health and mental health and how that could relate to our dental hygiene/care/condition”, “About CF in general and how CF has impacted our oral health”*.

The final theme that emerged and can be linked to dental anxiety as outlined in the previous section, is for dentists to be aware that adults with CF may be anxious about cross-infection risks. Respondents mentioned aspects such as “*cross-infection risks, especially infection risk from pseudomonas*”, *“clean lines”, “make sure all equipment is sterile for treatment”*.

## Discussion

Adults with CF can feel anxious regarding dental care which may result in irregular attendance and higher risk of dental disease. The most common concerns described by respondents of this study include dental anxiety, cross-infection concerns, and difficulty with the practicalities of treatment, for example, laying back in the dental chair.

33.8% of respondents said they were anxious regarding attending the dentist. This is a much higher proportion than the estimated 4% to 20% of people in the general population in developed countries who suffer from dental anxiety [31, 32]. It is also a higher proportion than a 1998 sample of people with special health care needs where 27.9% reported “fear/anxiety about dental visits” Ideally, levels of dental anxiety amongst the population would be decreasing year on year due to new therapies etc, therefore, this is a major concern. It is well established that dental anxiety is one of the most important barriers to dental care [33, 34]. Therefore, this study is important because it enables us to gain better understanding of what is causing the high levels of anxiety in adults with CF.

Examining the major themes regarding this anxiety, it can be seen there are dental-related concerns (the dentist themselves and treatment concerns) and patient-related concerns (problems with teeth and CF as a condition). Clinicians should seek to rectify the concerns over which they have control, i.e. being judgmental regarding patient’s teeth or need to stop for breaks. These authors recommend allocating extra time to patients with CF to enable clinicians to have an in-depth discussion regarding the patients’ previous medical and dental history, and concerns they may have regarding their teeth or regarding dental treatment. Good communication skills, and the feeling that patients are being listened to can result in a marked improvement in patient satisfaction and healthcare outcomes [35], therefore spending extra time to fully understand the CF patient’s medical background should help to improve their perception of dentists and dental treatment, and to alleviate their anxiety.

This study shows that adults with CF can themselves be concerned regarding the appearance of their teeth, and many believe the medication and diet related to CF has had a negative effect on their oral health. They have raised concerns about dentists’ lack of understanding on the condition and its management, and therefore, may be anxious about attending a dentist due to fear of being adversely judged. Adults with CF want dentists to understand that due to increased dietary needs, periods of ill-health and polypharmacy, they may have increased risk of oral disease. Therefore, dentists practising in areas with a relatively high proportion of PWCF, such as Ireland and the U.K., should educate themselves regarding the comorbidities and treatment modalities involved in this condition. More recommendations are given below.

One of the major points repeated was the need to sit the patient in an upright position due to a frequent need to cough, this is due to mucus build up or reflux, and is reported by almost every person with CF [36, 37]. Therefore, sitting the patient in an upright position during treatment should be accommodated if possible.

Dentists should also alleviate the patient’s concern regarding cross-infection control, by reassuring adults with CF that the strictest standards of cross-infection control are being adhered to.

A large number of respondents mentioned Covid-19 specifically as an area of concern, this is in-keeping with recent studies which showed that even in people without CF, “those with high Covid-19 fear were at least six times more likely to not visit the dentist” [38]. Similar to the previous comments about cross-infection control, it may be beneficial to allay PWCF’s fears regarding this by, for example, carrying out a Covid-19 test on any staff that may be in contact with the individual, wearing of FFP2 masks or similar.

This study is important as it provides the first insight into the attitudes of adults with CF towards dental treatment and gives recommendations for improving provision of care to these patients. There is a lot of overlap between what adults with CF want dentists to know, and what causes dental anxiety in them. Therefore, if dental practitioners are educated about CF and its repercussions and make small amendments to their practice based on these, it should make the dental experience less anxiety-inducing for adults with CF.

### Strengths and limitations

A limitation is the relatively small sample size. It should be noted there are 746 adults with CF in Ireland as of 2019 [39]; therefore, a response from approximately 9.5% of that population was obtained. As with any survey, there is a risk of sample selection bias, in that people who are anxious about attending the dentist may be more likely to self-select for the survey. Another limitation is the high level of education (67.6% completing third level) and of employment (60.6%) reported by the respondents. This is higher than the level of education (49% completing third level) and of employment (54%) seen in other Irish PWCF, as outlined in CF Ireland’s Independent Living report 2017 [40], and so may not be fully representative of the CF population as a whole. In future studies, it may be beneficial to collect data regarding the age of respondents and investigate if there is any age-related difference in dental concerns.

A major strength of this study is that it is the first study into the attitudes of adults with CF regarding dental treatment and should serve to guide dentists and other dental care providers in their management and treatment of this vulnerable cohort. This is the first opportunity adults with CF were given in order to voice their concerns or anxieties regarding dental treatment and it would be beneficial to compare it to other (non-Irish) CF populations to see if they have similar concerns.

Another strength is that the questionnaire was anonymous, therefore respondents were more likely to be honest with how they perceived the dentist and dental appointments. The fact that we employed the use of PPI (Patient and Public involvement) was a major benefit as a 2014 systematic review shows PPI has a a positive impact on all research/stages [41].

## Conclusions

A third of adults with CF reported anxiety about attending the dentist. Reasons for this included fear, embarrassment, cross-infection concerns and problems with treatment, especially being in the supine position. Dentists and dental care professionals should be aware of the impact that CF can have upon dental treatment and oral health care.

## Recommendations

Medical complexities faced by people with CF such as frequent and long-term antibiotic use, specific dietary requirements and periods of illness may lead to difficulty in maintaining meticulous oral hygiene. Dentists should be understanding of this and not chastise patients regarding their dental condition but instead give recommendations regarding how to improve this.

A thorough medical and social history should be carried out in order to improve patient care. Be aware that people with CF may not think to disclose the consumption of Oral Nutritional Supplements (ONS) in their medical history, and so should be asked about this separately. Preventative advice such as fluoride varnish application, fissure sealants or a fluoride mouth rinse may be advised if a patient is deemed at high risk of caries development, due to sugar-containing medicines, ONS, high calorie diets, presence of DDEs etc. Patients with CF-related diabetes should be warned re the potential risk of periodontal disease. The majority of PWCF take some form of inhaled medication and they should be advised to rinse out after use.

It goes without saying that the dental surgery and all instruments used must undergo the strictest levels of cross-infection control, however the patient with CF should be reassured that all standards are being met.

Dentists should endeavour to make the CF patient’s experience more comfortable by sitting the patient upright if possible, to facilitate mucus clearance. It may also be necessary to take a number of breaks in order to allow the patient to expectorate.

## Supplementary information

Appendices 1-4

## References

[1] Merjaneh L, Hasan S, Kasim N, Ode KL (2022). The role of modulators in cystic fibrosis related diabetes. J Clin Transl Endocrinol..

[2] Ronan NJ, Elborn JS, Plant BJ (2017). Current and emerging comorbidities in cystic fibrosis. La Presse Médicale.

[3] Ramos KJ, Smith PJ, McKone EF, Pilewski JM, Lucy A, Hempstead SE (2019). Lung transplant referral for individuals with cystic fibrosis: Cystic Fibrosis Foundation consensus guidelines. J Cyst Fibros..

[4] McBennett KA, Davis PB, Konstan MW (2022). Increasing life expectancy in cystic fibrosis: Advances and challenges. Pediatr Pulmonol..

[5] Scotet V, L’Hostis C, Férec C (2020). The Changing Epidemiology of Cystic Fibrosis: Incidence, Survival and Impact of the CFTR Gene Discovery. Genes (Basel).

[6] Taccetti G, Francalanci M, Pizzamiglio G, Messore B, Carnovale V, Cimino G (2021). Cystic fibrosis: Recent insights into inhaled antibiotic treatment and future perspectives. Antibiotics.

[7] Marquette M, Haworth CS (2016). Bone health and disease in cystic fibrosis. Paediatr Respiratory Rev..

[8] Robinson NB, DiMango E (2014). Prevalence of Gastroesophageal Reflux in Cystic Fibrosis and Implications for Lung Disease. Ann Am Thorac Soc..

[9] Bridges N (2013). Diabetes in cystic fibrosis. Paediatr. Respiratory Rev..

[10] Victoria C-B, Casilda O, Nuria P, José A-F, María G-O, José S-TF (2021). Oral nutritional supplements in adults with cystic fibrosis: effects on intake, levels of fat-soluble vitamins, and bone remodeling biomarkers. Nutrients..

[11] Coffey N, O’ Leary F, Burke F, Roberts A, Hayes M (2020). Periodontal and oral health status of people with cystic fibrosis: a systematic review. J Dent..

[12] Kinirons MJ (1992). The effect of antibiotic therapy on the oral health of cystic fibrosis children. Int J Paediatr Dent..

[13] Pawlaczyk-Kamieńska T, Borysewicz-Lewicka M, Śniatała R, Batura-Gabryel H (2019). Clinical evaluation of the dental hard tissues in an adult population with cystic fibrosis. Pol Arch Intern Med.

[14] Ferrazzano GF, Sangianantoni G, Cantile T, Amato I, Orlando S, Ingenito A (2012). Dental enamel defects in Italian children with cystic fibrosis: an observational study. Community Dent Health.

[15] Abu-Zahra R, Antos NJ, Kump T, Angelopoulou MV (2019). Oral health of cystic fibrosis patients at a north american center: A pilot study. Med Oral. Patol Oral Cir Bucal.

[16] Peker S, Mete S, Gokdemir Y, Karadag B, Kargul B (2014). Related factors of dental caries and molar incisor hypomineralisation in a group of children with cystic fibrosis. Eur Arch Paediatr Dent..

[17] Ferrazzano GF, Orlando S, Sangianantoni G, Cantile T, Ingenito A (2009). Dental and periodontal health status in children affected by cystic fibrosis in a southern Italian region. Eur J Paediatr Dent..

[18] Primosch RE (1980). Tetracycline discoloration, enamel defects, and dental caries in patients with cystic fibrosis. Oral Surg Oral Med Oral Pathol..

[19] Coffey N, O’ Leary F, Burke F, Roberts A, Howlett C, Plant B, et al. Oral nutritional supplements: sugar content and potential dental implications. Gerodontology. 2022;39:354–8. 10.1111/ger.12592 34569084

[20] Jagels AE, Sweeney EA (1976). Oral health of patients with cystic-fibrosis and their siblings. J Dent Res..

[21] Kinirons MJ (1989). Dental health of patients suffering from cystic fibrosis in Northern Ireland. Community Dent Health.

[22] Aps JK, Van Maele GO, Martens LC (2002). Caries experience and oral cleanliness in cystic fibrosis homozygotes and heterozygotes. Oral Surg Oral Med Oral Pathol Oral Radio Endod..

[23] Narang A, Maguire A, Nunn JH, Bush A (2003). Oral health and related factors in cystic fibrosis and other chronic respiratory disorders. Arch Dis Child.

[24] Chi DL, Rosenfeld M, Mancl L, Chung WO, Presland RB, Sarvas E (2018). Age-related heterogeneity in dental caries and associated risk factors in individuals with cystic fibrosis ages 6-20 years: A pilot study. J Cyst Fibros..

[25] Alkhateeb AA, Mancl LA, Presland RB, Rothen ML, Chi DL (2017). Unstimulated saliva-related caries risk factors in individuals with cystic fibrosis: a cross-sectional analysis of unstimulated salivary flow, pH, and buffering capacity. Caries Res..

[26] Dabrowska E, Błahuszewska K, Minarowska A, Kaczmarski M, Niedźwiecka-Andrzejewicz I, Stokowska W (2006). Assessment of dental status and oral hygiene in the study population of cystic fibrosis patients in the Podlasie province. Adv Med Sci..

[27] Hildebrandt T, Zawilska A, Trzcionka A, Tanasiewicz M, Mazurek H, Świętochowska E (2020). Estimation of proinflammatory factors in the saliva of adult patients with cystic fibrosis and dental caries. Medicina..

[28] Alfaraj A, Halawany HS, Al-Hinai MT, Al-Badr AH, Alalshaikh M, Al-Khalifa KS (2021). Barriers to dental care in individuals with special healthcare needs in qatif, saudi arabia: a caregiver’s perspective. Patient Prefer Adherence.

[29] Coffey N, O’ Leary F, Burke F, Plant B, Roberts A, Hayes M. Self-reported Dental Attendance, Oral Hygiene habits and Dietary Habits of Adults with Cystic Fibrosis in the Republic of Ireland. Special Care Dent. 2022;1–8. 10.1111/scd.12773 36029268

[30] Braun V, Clarke V (2006). Using thematic analysis in psychology. Qualitative Res. Psychol..

[31] Locker D, Thomson W, Poulton R (2001). Psychological disorder, conditioning experiences, and the onset of dental anxiety in early adulthood. J Dent Res..

[32] Sukumaran I, Taylor S, Thomson WM (2021). The prevalence and impact of dental anxiety among adult New Zealanders. Int Dent J..

[33] Freeman R (1999). Barriers to accessing dental care: patient factor. Br Dent J..

[34] Hill KB, Chadwick B, Freeman R, O’Sullivan I, Murray JJ (2013). Adult Dental Health Survey 2009: relationships between dental attendance patterns, oral health behaviour and the current barriers to dental care. Br Dent J..

[35] Berman AC, Chutka DS (2016). Assessing effective physician-patient communication skills: “Are you listening to me, doc?”. Korean J Med Educ..

[36] Fathi H, Moon T, Donaldson J, Jackson W, Sedman P, Morice AH (2009). Cough in adult cystic fibrosis: diagnosis and response to fundoplication. Cough..

[37] Penketh A, Wise A, Mearns M, Hodson M, Batten J (1987). Cystic fibrosis in adolescents and adults. Thorax..

[38] González-Olmo MJ, Delgado-Ramos B, Ortega-Martínez AR, Romero-Maroto M, Carrillo-Díaz M (2022). Fear of COVID-19 in Madrid. Will patients avoid dental care?. Int Dent J..

[39] Ireland CFRI. CF Annual Report 2018 2019 [Available from: https://www.cfri.ie/docs/annual_reports/CFRI2018.pdf.

[40] Ireland CF. Independent Living and Cystic Fibrosis Report. 2018 March 2018.

[41] Brett J, Staniszewska S, Mockford C, Herron-Marx S, Hughes J, Tysall C (2014). A systematic review of the impact of patient and public involvement on service users, researchers and communities. Patient.

