# Supplementary material for: Oral care considerations for people with cystic fibrosis: a cross-sectional qualitative study
Source: BDJ Open. 2023 Mar 11;9:11. doi: 10.1038/s41405-023-00136-w (PMC10008013; doi:10.1038/s41405-023-00136-w)
Supplement: Supplementary file 1 — Appendices 1-4 [file 41405_2023_136_MOESM1_ESM.pdf]

Appendix I: Questionnaire

1. Do you consent to participate in this study? Yes/No
2. Are you over the age of 18? Yes/No

Note: This survey is only for people aged 18 and over.

3. Male __ Female ___
4. How many natural teeth do you have? No natural teeth / 1-9 teeth /10-19 teeth/ 20 or more teeth
5. During the past 12 months, did your teeth or mouth cause any pain or discomfort? Y/N/Don't know
6. Are you a regular attender? If so how regular- every 3, 6 or 12 months?
7. What was the reason for your last visit to the dentist? Routine exam/pain/treatment etc.
8. Are you happy with the appearance of your teeth? Yes/No/Don't know
   If no, please explain _____________
9. In relation to CF, are you anxious/ worried attending your dentist? Yes/No/Don't know. If yes, why?
10. Do you make the dental receptionist aware of your CF status when scheduling an appointment? Yes/No/Don't know
11. Are there any precautions you take when attending your dentist? E.g schedule appointment for first thing in the morning/ minimise contact with other patients? Yes/No/Don't know
12. Do you believe that CF has impacted on your oral health in any way?e.g. too tired to brush your teeth during bouts of illness etc.

In relation to CF, are you anxious/ worried attending your dentist?

Why do you feel this way regarding attending the dentist?

What do you feel is important for dentists to know about the condition?

13. Are you employed? Full Time employment or part time employment?
14. Are you in receipt of a social welfare benefit? If so, what one- Disability Allowance, Illness Benefit etc.
15. What level of education have you completed? No formal schooling/Less than primary school/Primary school completed/ Secondary school completed/High school completed /College or university completed/Postgraduate degree

Appendix II: Do you believe CF has an impact on your oral health? If yes, what impact has it had?

**Medication**	Caused vomiting<br>some treatments cause vomiting, cannot brush due to vomiting<br>when on some meds-nausea due to tb<br>meds causing discolouration<br>meds causing discolouration<br>after IVs-more plaque<br>dry mouth from meds<br>dicolouration<br>staining and discolouration<br>inhaled meds weakened my teeth<br>tired-need energy, go for sugar snack<br>tablets and nebs causing problems
**A/bs**	Cause staining<br>Eroding teeth<br>Cause discolouration<br>Caused needing many fillings<br>Cause lost enamel

	sugary diet, sugary meds
**Tiredness**	Tired<br><br>too tired<br><br>tiredness, less energy at night<br><br>on IVs weakness and ill-no energy<br><br>when sick, too much energy to brush<br><br>no energy to brush teeth, “I remember having to ask my mum to brush them for me.”<br><br>too tired<br><br>tiredness and depression<br><br>too tired to brush and floss<br><br>too tired
**Diet**	dicolouration<br><br>eat a lot of sweet to get calories in<br><br>addicted to sugar<br><br>poor taste so eat strong flavours with high fat and sugar<br><br>eating jellies and OJ to treat hypos since developing CFRD

**CF**	tummy issues causing bad breath and<br><br>erosion<br><br>missed appts due to being unwell<br><br>unable to brush teeth when unwell

Appendix III

Thematic analysis: Are you anxious regarding attending the dentist? If yes, why?

Theme

**Dentist-related issues**	"I have previously been treated with lack of understanding from dentists/hygienists"<br>"fear I will be judged by the dentist"<br>"fear of dentists"<br>"I am always in trouble when I attend"
**Cross-infection concerns**	'Cross-infection due to sink at dentist chair"<br>"risk of infection- I did not feel this way before but I do now in light of Covid-19"
**Concerns regarding dental treatment**	"I find it difficult to be in a flat position as it affects my breathing. It's also very difficult not to cough"<br>"Chair tilted back, might induce a cough when dentist working closely"<br>"fear of extractions and the injections required into gums"
**Problems with teeth**	"embarrassed about my teeth"

	"I feel my teeth are going to break soon sometimes"
**CF related problems**	"Feel sick enough without there being another thing wrong with my teeth"

Appendix IV: *What do you think is important for the dentist to know about CF?*

**Theme**	**Select Quotations**
**Practicalities of dental tx**	That it is often difficult for people with cf to lie down and not cough for long periods of time.<br>that we need to be in an upright condition.<br>It would be great to do the treatment upright and have multiple breaks<br>When i need to cough he stops, not lying too flat back in chair<br>Possible breathing difficulty when in chair if patient has infection.<br>*My dentist is very good and tilts chair upright and also pauses when I indicate I need to cough. It slows process but get us very understanding and allows time*<br>That I can't lie flat or sometimes breathe through my nose quick enough<br>*Extra breaks may be needed, my dentist/hygienist are good when it come to that!*<br>Difficult to lie in one position for a long period of time, may need breaks for coughing<br>Sometimes it is hard for people with cf to sit in the dental chair in the drainage position as it can cause coughing fits

	Would have liked option to sit more upright during prolonged treatment, felt this was where I was exposed to risk of aspiration<br>In case I cough during the procedure.<br>Position of chair. Breaks needed for coughing.<br>Lying back can induce bronchospasm
**Impact of medication/tx/diet**	All the nebs we inhale we take could definitely have a part in destroying our teeth<br>Affects from antibiotics / diet<br>The full extent of the treatment required that may impact the teeth, gums and tongue. That some medication is delivered via inhaler and nebuliser.<br>before lecture on oral hygiene sweets etc CF patients need to maintain weight.<br>More about the impact of our long term medication has had on our teeth and about cf in general<br>CF dietary requirements,<br>Impacts of antibiotics on tooth heath<br>discolouration from medication<br>The effect different meds have on oral hygiene
**Impact of CF**	About cf in general and how cf has impacted out oral health

	Antibiotic use in CF, dietary requirement in CF, cross-infection risks in CF and CF vulnerability to pseudomonas especially<br>How badly it affects your teeth<br>Yes due to calcium deficiency<br>pre and post transplant care.<br>To have an understanding really would be a good start.<br>All of it and the impact it has on our health and mental health and how that could relate to our dental hygiene/care/condition<br>Infection ,low immune system
**Cleanliness/sterility of dental surgery**	Cross-infection of patients with CF<br>Going forward the importance of ppe when a cf patient is undergoing treatment<br>Clean lines, cross-infection<br>That of a member of staff has a cold,cough or flu I will not attend.<br>cross-infection risks, especially infection risk from pseudomonas

<u>Abbreviations</u>

CF- Cystic Fibrosis

DDE- Developmental Defects of Enamel

PWCF- People with CF
